# Identification of putative essential protein domains from high-density transposon insertion sequencing

**DOI:** 10.1038/s41598-022-05028-x

**Published:** 2022-01-19

**Authors:** A. S. M. Zisanur Rahman, Lukas Timmerman, Flyn Gallardo, Silvia T. Cardona

**Affiliations:** 1 Department of Microbiology, University of Manitoba, Winnipeg, MB Canada; 2 Department of Computer Science, University of Manitoba, Winnipeg, MB Canada; 3 Department of Medical Microbiology and Infectious Diseases, University of Manitoba, Winnipeg, Canada

**Keywords:** Bacterial genomics, Bacterial genetics, Sequence annotation, Computational biology and bioinformatics, Microbiology, Molecular biology

## Abstract

A first clue to gene function can be obtained by examining whether a gene is required for life in certain standard conditions, that is, whether a gene is essential. In bacteria, essential genes are usually identified by high-density transposon mutagenesis followed by sequencing of insertion sites (Tn-seq). These studies assign the term “essential” to whole genes rather than the protein domain sequences that encode the essential functions. However, genes can code for multiple protein domains that evolve their functions independently. Therefore, when essential genes code for more than one protein domain, only one of them could be essential. In this study, we defined this subset of genes as “*e*ssential *d*omain-*c*ontaining” (EDC) genes. Using a Tn-seq data set built-in *Burkholderia cenocepacia* K56-2, we developed an in silico pipeline to identify EDC genes and the essential protein domains they encode. We found forty candidate EDC genes and demonstrated growth defect phenotypes using CRISPR interference (CRISPRi). This analysis included two knockdowns of genes encoding the protein domains of unknown function DUF2213 and DUF4148. These putative essential domains are conserved in more than two hundred bacterial species, including human and plant pathogens. Together, our study suggests that essentiality should be assigned to individual protein domains rather than genes, contributing to a first functional characterization of protein domains of unknown function.

## Introduction

**Figure 1:**
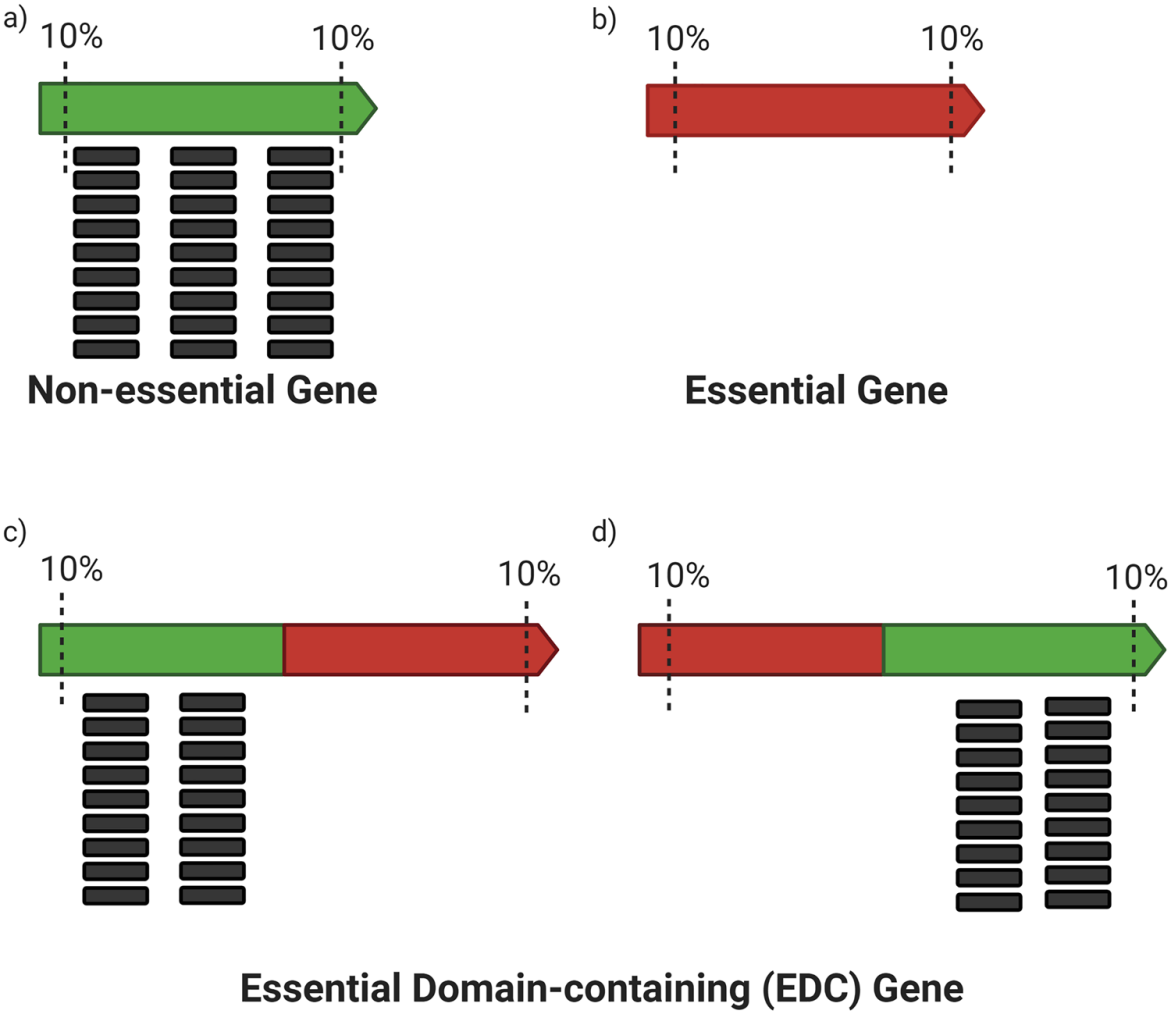
Schematics of Tn-Seq reads mapped to the insertion sites in non-essential (**a**), essential (**b**), and essential domain-containing (EDC) genes (**c**–**d**). The number of transposon insertions related to the length of the gene (minus the non-informative 10% towards the 5′ and 3′ ends) is quantified and used to classify genes as non-essential (**a**), or essential (**b**) according to the relative number of reads mapped to that gene. Tn-seq analysis may miss EDC genes which are essential genes that contain an essential domain not spanning throughout the whole length of the gene (**c**–**d**). Genes are represented by arrows. Tn-seq reads that map to regions of those genes are represented by black boxes. Essential and non-essential regions are colored in red and green, respectively.

A first step when characterizing gene function should be asking whether a given gene encodes an essential cellular function, whether the gene is necessary for the survival of the organism. A widely accepted method to identify essential genes in bacteria is high-density transposon mutagenesis, followed by Illumina-sequencing of the transposon insertion junctions (Tn-seq)^1^. During Tn-seq, transposon mutant cells are pooled and grown in optimal conditions, allowing cells with a transposon insertion located in a non-essential element to survive. Cells with a transposon insertion in an essential element should be lost or depleted from the population. When transposon insertions are identified by Illumina sequencing, read counts per gene in the central 70–90% of the open reading frame (disruptive insertions) are normalized by gene length and used to predict essentiality. 5–15% sequences from the 3′ and 5′ ends are usually removed from the analysis, as insertions within the terminal regions are likely non-disruptive^2–5^. While disrupted genes are regarded as “non-essential,” the method yields a list of putative essential genes as those with zero or very few mapped reads (Fig. 1a, b)^3^.

Another step towards identifying gene function is the annotation of the protein domains encoded by genes. Protein domains are functional or structural units that can fold, evolve, and function independently. Homology-based protein domain prediction and function assignment are effective starting points for understanding protein function, even when diverse protein architectures add complexity to functional annotations^6,7^. While domain databases such as Pfam^8^ and InterPro^9^ aim to provide maximum sequence coverage to predict protein domain identity, approximately 30% of all domains listed in these databases (Pfam 33.1 and InterPro 81.0) are ‘domains of unknown function (DUFs).’ Single DUFs are usually predicted to span through functionally uncharacterized proteins. However, studies suggest that at least some of these proteins may contain more than one domain^10,11^.

While robust and comprehensive, very few Tn-seq studies^12–14^ consider that genes may encode for more than one protein domain. Tn-seq analysis may classify a gene as “non-essential” due to the presence of transposon insertions in a non-essential coding region, despite the gene coding for a second domain not spanning through the whole gene length that might be essential^3,15,16^. We operationally defined this subclass of essential genes as “*e*ssential *d*omain-*c*ontaining” (EDC) genes (Fig. 1c, d) and present a computational pipeline to identify them in a Tn-seq dataset built-in *Burkholderia cenocepacia* K56-2^17^. Unlike the previously reported methods, our method does not require in-depth understanding of computational platforms and generates a list of candidate EDC genes. By analyzing biases in transposon density in genes previously identified as “non-essential”, we found 40 genes where the encoded proteins contained putative essential and non-essential domains. Using a CRISPR Interference (CRISPRi)^18^ platform we developed for *Burkholderia*^19^, we experimentally confirmed growth defects, representing the loss of a putative essential function, in 27 EDC gene knockdowns. The identified EDC genes include ten encoding known multidomain proteins and two entirely uncharacterized genes encoding different N-terminal DUFs, demonstrating the utility of the approach. This study highlights that gene essentiality depends on the function of individual protein domains rather than entire proteins.

## Results

### Identification of EDC genes from Tn-seq data

**Figure 2:**
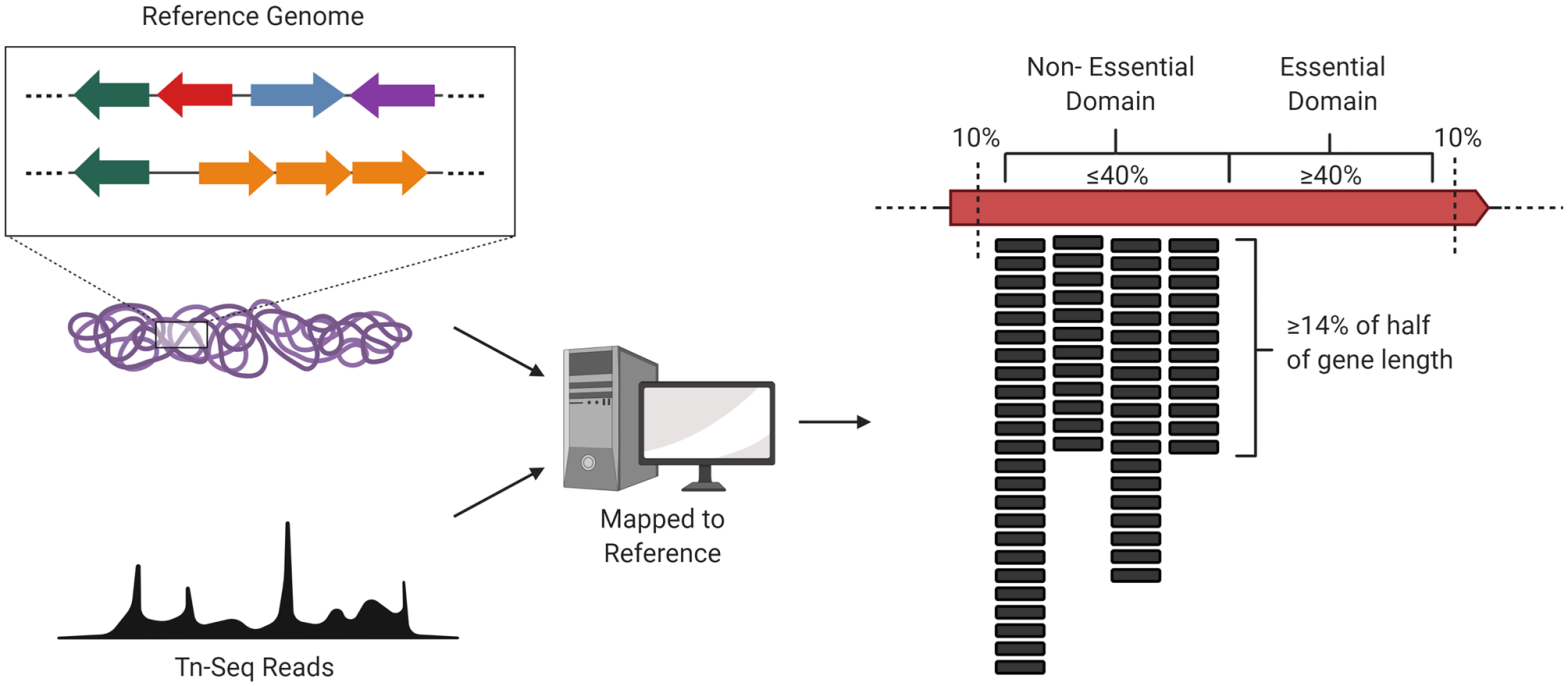
Identification of putative essential domain-containing (EDC) genes from a Tn-seq dataset. Tn-Seq reads are first mapped to the reference genome. A custom-built script identifies genes with biased location of transposon insertions towards one half of the gene. The script parameters “min ratio’ and “min reads” were set such that genes were selected when (i) a half-region of that gene (at least 40% of the total gene length) showed no insertions (min ratio = 0), and (ii) the other half contained mapped reads in at least 14% of that gene-half length (min reads = 0.14) (see Supplementary Fig. 1and Material and Methods for details). Reads mapping to each 5′ or 3′ 10% end of the gene were discarded from the analysis.

To identify EDC genes in *B. cenocepacia* K56-2, we built a custom script that used our previous Tn-seq data^17^ to select genes that (i) were not previously found to be essential in *B. cenocepacia* K56-2^17^, and (ii) had an asymmetric distribution of transposon insertions (Fig. 2). The script split each gene into two equal parts and selected genes with reads in only one region to identify genes with transposon insertion biases. We worked under the assumption that (i) each half could represent one functional domain and (ii) one of the domains may be essential while the other may not. We arbitrarily set the parameters “min ratio” and “min reads” to 0 and 0.14, respectively (see Material and Methods and Supplementary Fig. 1). These settings looked for genes that had zero reads at one end, while the number of reads in the non-empty end was at least 14% of that region's length. For example, if a section of a gene was 100 bp in length, it would require at least 14 reads mapped to that section to be considered non-essential. With these settings, the script produced an extensive list of 178 candidate EDC genes (Supplementary Table 1).

### Bioinformatic analysis of the candidate EDC genes

**Figure 3:**
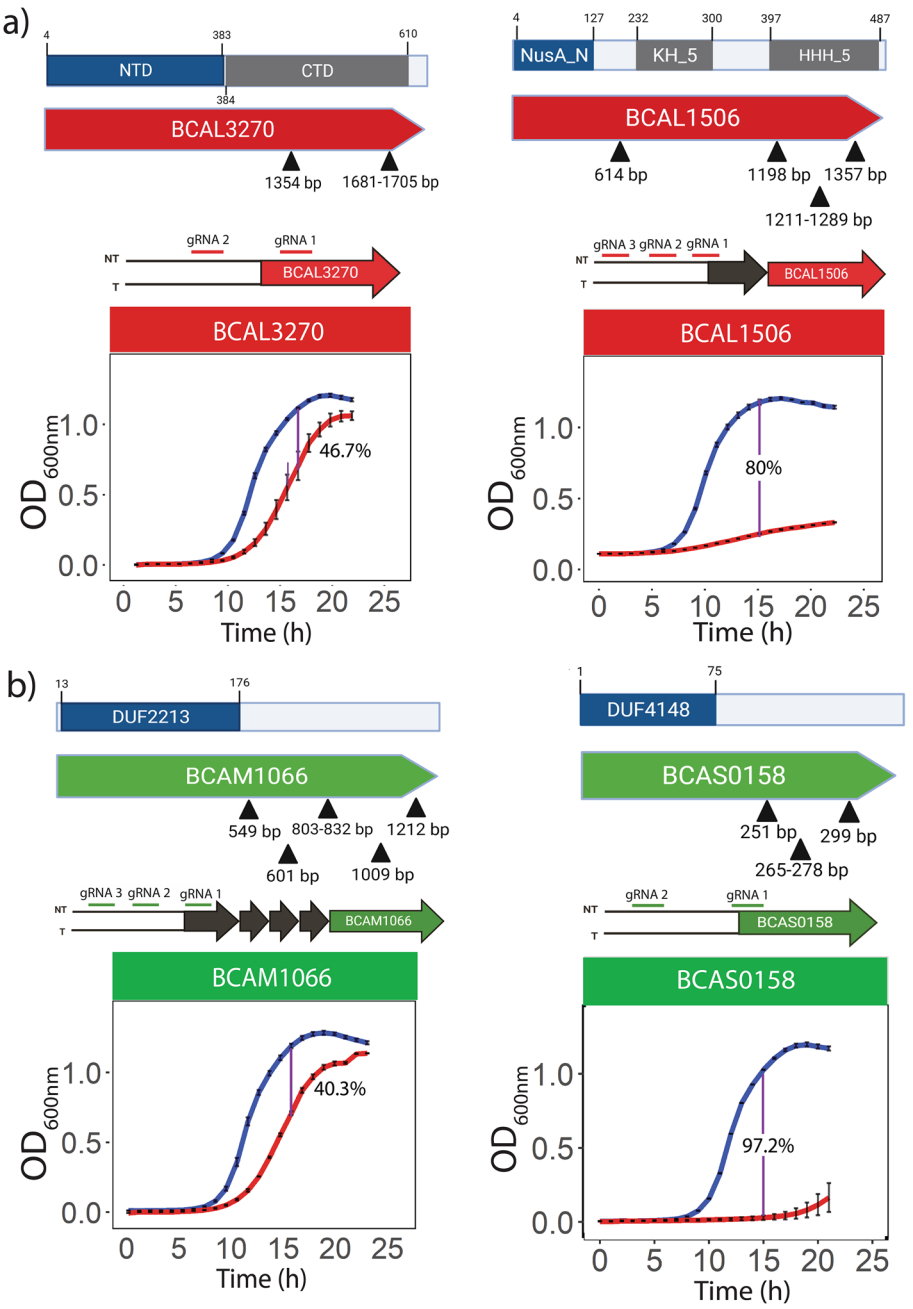
Biased transposon insertion identifies putative essential domains of uncharacterized hypothetical proteins. Tn-seq reads from^17^ were mapped to the *B. cenocepacia* K56-2 genome and predicted to contain essential domains. (**a**) The script identified the well characterized essential N-terminal domains of DnaK (BCAL3270) and NusA (BCAL1506). Their respective CRISPRi mutants demonstrated a conditional growth defect. (**b**) Two uncharacterized genes BCAM1066 (WQ49_RS16145) and BCAS0158 (WQ49_RS10495) contain the Pfam domains DUF2213 (PF09979) and DUF4148 (PF13663), respectively, at the N-terminal end. The Tn-seq reads map to the C-terminal end of these genes, demonstrating the essentiality of DUF2213 and DUF4148. Putative essential domains are highlighted in blue. Black triangles represent the transposon insertion sites. Numbers on top of the domains denote amino acid sequence positions. Blue and red lines in the growth curves (**a** and **b**) represent growth in the absence and presence of rhamnose, respectively. Growth curves are shown for the most efficient sgRNAs. Growth curves values are the average of three independent biological replicates. Error bars indicate mean ± SD.

We reasoned that if EDC genes contained essential protein domains, then the essential protein domains may be encoded by essential genes in at least some other bacteria. We then searched for essential ortholog genes of the 178 candidate EDC genes by BLASTx searches against the ‘Database of Essential Genes (DEG)^20^ using 50% sequence alignment and 30% sequence identity as the cut-off. We found that 40 of the 178 genes had orthologs annotated as ‘essential” in other bacterial species. We wished to interrogate the domains encoded by these 40 genes using UniProt^21^ based on InterPro domains^9^. InterPro predicts the domain information by matching the protein or nucleic acid sequences against the member databases (collectively known as InterPro consortium) to identify ‘signatures’ associated with known domains. Thus, the InterPro prediction relies on the availability of sequence characterization and annotation. This analysis showed that from the 40 candidate EDC genes predicted to be essential by homology with other essential genes, 10 genes encoded multidomain proteins, and 7 of them were well-characterized, such as the N-terminal domain of DnaK and NusA (Fig. 3a). The remaining genes were predicted to have one single annotated domain (19 genes) that did not span the whole gene-length or encoded uncharacterized proteins (11 genes) (Supplementary Table 2). All 40 genes had transposon insertions located in one half of the gene, showing that the script was able to identify genes with biased transposon insertions (Supplementary Fig. 2). Taken together, these results suggest that the identified genes could be essential due to the presence of essential protein domain orthologues. Notably, 17 DNA regions were identified as coding for new putative essential protein domains (Table 1).

**Table 1:** Putative essential genes and domains identified based on biased transposon insertions.

K56-2 locus tag	Homolog J2315 locus tag	Product name	Function	Reads at 5′ half	Reads at 3′ half	Identified putative essential domain
WQ49_RS00050	BCAL3469	Cell division protein FtsL	Essential cell division protein	0	23	Domain (FtsL)
WQ49_RS00770	BCAL3328	NUDIX hydrolase	Nucleoside-diphosphatase	0	49	Domain (Nudix hydrolase)
WQ49_RS00885	BCAL3305	Preprotein translocase subunit YajC	Secretase/insertase	21	0	New
WQ49_RS01035	BCAL3270	DnaK	Chaperone	0	227	N-terminal Domain
WQ49_RS02920	BCAM1451	Hypothetical protein	Unknown	43	0	New
WQ49_RS03160	BCAM1502	Hypothetical protein	Unknown	59	0	New
WQ49_RS03550	QU43_RS62245	Hypothetical protein	Unknown	33	0	New
WQ49_RS03805	BCAM1624	MaoC family dehydratase	MaoC-like dehydratase	46	0	New
WQ49_RS04450	BCAM1749	Hypothetical protein	Unknown	17	0	New
WQ49_RS07360	BCAM2338	Glycosyl transferase family 1	UDP-glycosyltransferase	0	152	Domain (Glyco_transf_28)
WQ49_RS07395	QU43_RS66100	Hypothetical protein	Unknown	0	58	New
WQ49_RS09185	BCAS0417	Cytochrome biogenesis protein CcdA	Electron transfer	0	38	New
WQ49_RS10495	BCAS0158	hypothetical protein	Unknown	0	34	Domain (DUF4148)
WQ49_RS11915	BCAL0324	TatB	Protein Transmembrane transporter	0	57	Domain (TatA_B_E)
WQ49_RS12045	BCAL0298	Thiamine biosynthesis protein ThiS	Thiamine biosynthesis protein ThiS	0	50	Domain (ThiS)
WQ49_RS12280	BCAL0250	50S ribosomal protein L18	Structural constituent of ribosome	0	65	Domain (Ribosomal_L18p)
WQ49_RS12305	BCAL0245	RplX	Structural constituent of ribosome	20	0	Domain (L24-Pfam)
WQ49_RS12315	BCAL0243	30S ribosomal protein S17	Structural constituent of ribosome	0	64	New
WQ49_RS12365	BCAL0233	RpsJ	Structural constituent of ribosome	0	25	New
WQ49_RS16145	BCAM1066	Hypothetical protein	Unknown	0	425	Domain (DUF2213)
WQ49_RS18705	BCAM0549	Molecular chaperone GroES	Chaperone	0	21	Domain (Cpn10)
WQ49_RS22170	BCAM2699	alpha/beta hydrolase	Putative hydrolase	120	0	Domain (Abhydrolase_3)
WQ49_RS23945	BCAL0558	Cca	3′-Cytidine-cytidine-tRNA adenylyltransferase	0	79	Domain (PolyA Polymerase)/Domain (Binding)
WQ49_RS24070	BCAL0585	Hypothetical protein	Unknown	0	23	new
WQ49_RS25525	BCAL0878	FmdB family transcriptional regulator	Regulatory activity	0	30	Domain (CxxC_CXXC_SSSS)
WQ49_RS25680	BCAL0909	16S rRNA maturation RNase YbeY	Endoribonuclease activity	68	0	Domain (UPF0054)
WQ49_RS26625	BCAL2715	RpmG	Structural constituent of ribosome	0	31	Domain (Ribosomal_L33)
WQ49_RS27920	BCAL2334	NADH-quinone oxidoreductase subunit K	NADH dehydrogenase	0	21	Domain (Oxidored_q2)
WQ49_RS28635	BCAL2199	Fe–S cluster assembly transcriptional regulator IscR	DNA-binding transcription factor	39	0	Domain (Rrf2)
WQ49_RS29230	BCAL2091	30S ribosomal protein S2	Structural constituent of ribosome	0	86	Domain (Ribosomal_S2)
WQ49_RS30770	BCAL1788	Biopolymer transporter ExbD	Transmembrane transporter	0	47	Domain (ExbD)
WQ49_RS31735	NA	Hypothetical protein	Unknown	0	42	New
WQ49_RS31805	BCAL1585	Transcriptional regulator	DNA binding	44	0	New
WQ49_RS32210	BCAL1506	NusA	DNA-binding transcription factor	0	93	Domain (NusA_N)
WQ49_RS32225	BCAL1503	SMC-Scp complex	Cell Division/chromosome separation	0	94	Domain (SMC)
WQ49_RS32625	BCAL1424	ABC transporter	ATPase	63	0	New
WQ49_RS34660	BCAL0990	50S ribosomal protein L32	Structural constituent of ribosome	27	0	New
WQ49_RS34895	BCAL2925	50S ribosomal protein L19	Structural constituent of ribosome	0	26	Domain (Ribosomal_L19)
WQ49_RS35060	BCAL2958	Membrane protein	Porin activity	43	0	Domain (OmpA)
WQ49_RS03390	BCAM1545	LuxR family transcriptional regulator	DNA binding	251	0	Domain (HTH luxR-type)

### CRISPRi knockdowns of EDC genes show growth defects

To phenotypically characterize the effect of knocking down EDC genes, we used CRISPR interference or CRISPRi^19^ to create knockdown mutants of the genes of interest. CRISPRi comprises a chromosomally integrated *dcas9* under the control of a rhamnose-inducible promoter and plasmid-borne sgRNA driven by a constitutively active synthetic promoter, P_J23119_^19^. Simultaneous expression of *dcas9* and a target-specific sgRNA allows the dCas9 to bind the target DNA region and, thus, sterically interfere with transcription by RNA polymerase^18,19^. To inhibit the expression of the candidate genes, we designed two sgRNAs against each of the candidate genes targeting the start codon and adjacent region on the non-template strand (Supplementary Fig. 3a,c). For phenotypic characterization, we grew the cells in LB with and without rhamnose. Upon induction of dCas9 with rhamnose, 27 out of the 40 candidate genes showed at least 25% growth inhibition relative to the uninduced condition (Supplementary Fig. 3b,d).

### DUF2213 and DUF4148 appear to be essential domains

**Figure 4:**
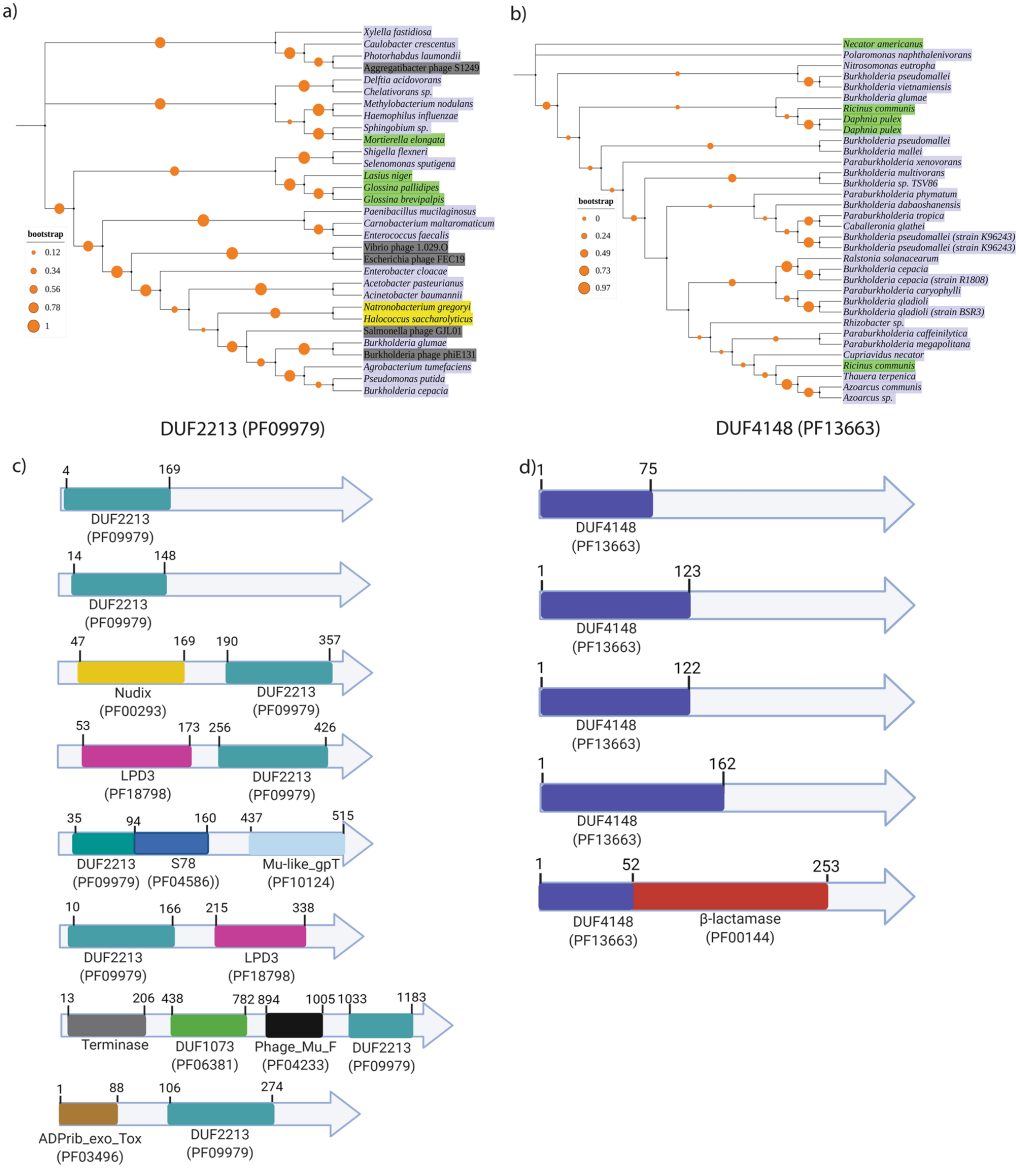
Phylogenetic trees with taxonomic information of DUF2213 (PF09979) and DUF4148 (PF13663) and domain architectures of proteins containing these domains. (**a**–**b**) Phylogenetic trees of DUF2213 (**a**) and DUF4148 (**b**) across the species with taxonomic annotations. DUF2213 is widely distributed within bacterial, archaeal, phage and eukaryotic species, whereas DUF4148 is mostly distributed in bacteria (primarily in Proteobacterial species). Trees shown here are the majority rule consensus trees. Taxonomic annotations were labelled based on NCBI taxonomy database. Representative bacterial, archaeal, phage and eukaryotic species are highlighted in lilac, yellow, grey and green, respectively. The orange circles on the branches represent the bootstraps values. (**c**)–(**d**) Domain architectures of proteins containing DUF2213 (**c**) and DUF4148 (**d**) across species. Numbers on top of the domains in (**c**) and (**d**) represent amino acid sequence positions.

The presence of DUFs is a common feature of hypothetical or uncharacterized proteins. To initiate functional characterization of DUFs, we focused on two genes containing DUF-coding sequences, which their respective CRISPRi mutants demonstrated a conditional growth defect (Fig. 3b). WQ49_RS16145 (BCAM1066) and WQ49_RS10495 (BCAS0158) contain DUF2213 (Pfam accession PF09979) and DUF4148 (Pfam accession PF13663), respectively at the N-terminal end of the proteins (Fig. 3b). BLAST searches of BCAM1066 and BCAS0158 genes as a query against the DEG^20^ showed that BCAM1066 (WQ49_RS16145) had 30% sequence similarity with *lysK* (B8GXH3) from *Caulobacter crescentus,* and BCAS0158 (WQ49_RS10495) had a 52% sequence identity with a predicted amino acid permease (BPSS1112) from *Burkholderia pseudomallei* K96243 (data not shown). Mining of the Pfam database (https://pfam.xfam.org/) showed that these DUFs are well conserved across the bacterial species: DUF2213 is present in 209 bacterial species, including bacterial pathogens (*Acinetobacter baumannii*, *Enterobacter cloacae*, *Haemophilus influenzae, Burkholderia cepacia, Shigella flexneri*), plant pathogens (*Agrobacterium tumefaciens*), and biotechnologically relevant species (*Pseudomonas putida)* (Fig. 4a and Supplementary Table 5). DUF4148 is found in 204 bacterial species, primarily in *Burkholderia* species (i. e *Burkholderia cepacia, Burkholderia mallei, Burkholderia vietnamiensis*) and plant pathogens such as *Ralstonia solanacearum* (Fig. 4b; Supplementary Table 5). DUF2213 is also present in many phage-related proteins (Fig. 4a). Eight unique domain architectures were observed for proteins containing DUF2213 and five for DUF4148 (Fig. 4c, d). DUF2213 is associated with another essential domain PF00293, a NUDIX hydrolase (Fig. 4c). In other proteins, DUF2213 is associated with the LPD3 domain (PF18798) and DUF1073 (PF06381) which is also conserved across bacterial species^11^ (Fig. 4c). On the other hand, Pfam analysis of DUF4148 shows that DUF4148 differs in domain length among species and is associated with the Pfam domain PF00144, known to confer resistance against β-lactams (Fig. 4d)^22^. Nonetheless, the encoded N-terminus was highly conserved, suggesting that it is functionally significant. The Pfam-based analysis of species distribution also revealed that DUF2213 is present in six eukaryotic species (five metazoans and one fungal species), whereas DUF4148 is present in five eukaryotic species (three viridiplantae species and two metazoan species). The widespread distribution of these DUFs indicates the functional importance of these putative essential domains, creating an impetus for further characterization.

## Discussion

A first step in the functional characterization of genes is performed through gene deletion or gene silencing and growth phenotype characterization. For genes that encode multidomain proteins performing multiple functions driven by the activity of their individual domains^23^, the function assigned to a gene could indeed correspond to one of its encoded protein domains and not to the whole protein. That is the case of essential genes identified by Tn-seq^1^. In standard Tn-seq analysis the condition of essentiality is assigned to genes and not to encoded domains, resulting in incorrect classification of many essential genes as non-essential. Rather, the essentiality assignment pipeline should be revised to analyze the essentiality of encoded individual protein domains^24^. Indeed, essentiality can be assigned to individual domains of a multidomain protein rather than the entire protein^15,16^. In this work, we defined as essential-domain-containing (EDC) genes those genes that encode more than one protein domain, with one of the domains coding for an essential function. By analyzing a Tn-seq dataset^17^ for transposon insertion biases, we show that standard Tn-seq analysis pipelines may miss EDC genes, whose detection often requires either manual curation or additional considerations^25^.

We validated our approach by identifying genes encoding previously characterized multidomain essential proteins in which the essential function is assigned to one single domain. For instance, our analysis of biases in the Tn-seq dataset showed that the gene region coding for the N-terminal domain of NusA^26^ is sufficient to mediate the essential function, in agreement with previous work^27^. Similarly, the *B. cenocepacia* K56-2 *dnaK* gene was previously defined as non-essential^17^; however, we found that the Tn-seq reads mapped onto *dnaK* were biased toward the C-terminal domain (CTD), suggesting that only the NTD is necessary for its essential function. (Fig. 3b; Supplementary Fig. 2). DnaK is a multidomain protein and a master regulator of the chaperone network^28^. DnaK comprises an N-terminal ATPase domain (NTD) and a C-terminal substrate-binding domain (CTD)^28^. Perturbations either within the NTD that leads to the abrogation of the ATPase activity or within the conserved linker peptide that impairs the interdomain mechanistic interaction abrogate the in vivo activity of DnaK^29,30^.

While 14 EDC genes that demonstrated a growth defect when knocked down code for proteins annotated to have a single domain, none of these domains span the entire gene, and transposon insertions are only mapped to the annotated domain (Supplementary Fig. 2). Thus, it is possible that the remaining regions code for novel domains that perform the essential biological functions independently of the adjacent sequences. Indeed, multidomain proteins that are involved in direct protein–protein interactions are more often detected as essential than proteins with a single domain^15^, hinting towards the functional contribution of individual domains within a protein complex. However, it should be noted that the presence of multiple domains in an essential protein does not necessarily mean that the protein is composed by essential and non-essential domains. An example is the *Bacillus subtillis* SMC, a multidomain essential protein involved in chromosomal segregation^31,32^.

We demonstrated a conditional growth defect in 27 out of 40 CRISPRi mutants of EDC genes. It remains a possibility that the sgRNAs designed for CRISPRi-mediated gene silencing of the remaining 13 genes were not efficient in target binding, thus yielding no growth defect. CRISPRi is more effective in blocking transcription initiation than elongation, and is the most efficient in silencing gene expression when promoter regions are targeted with gRNAs^18,33–35^. However, as promoter regions for *B. cenocepacia* genomes remained largely unannotated we targeted translation start sites. It remains to be investigated whether targeting the promoter region to block the transcription initiation rather than elongation might yield conditional a growth phenotype in the remaining 13 genes.

Eighteen of the 27 EDC genes CRISPRi mutants that demonstrated a conditional growth defect are in an operon (Supplementary Fig. 3). It is possible then that due to the polar effect of CRISPRi, the observed growth defect could result from the transcriptional silencing of any other gene(s) in the same operon. However, we consider this possibility unlikely. These genes (other than the candidate gene in the operon) had transposon insertions greater than the defined threshold in the script across the entire genes (data not shown), suggesting that they are dispensable. The only exceptions are BCAL0245 and BCAL0250, where both genes are located in the same operon (Supplementary Fig. 3). Thus, it remains a possibility that observed growth defect could be due to transcriptional silencing of either or both the genes. A large portion of the protein domains that lack functional assignment can be grouped within the DUF category. DUFs are members of ever-increasing uncharacterized protein families; they are the object of experimental and computational efforts towards their functional characterization^10,36–38^. Determining if a DUF is essential is among the first steps in functional characterization. In this study, we focused on two EDC genes that encode putative essential DUFs: DUF2213 and DUF4148. Both domains have a high degree of conservation across diverse phyla, which highlights their biological relevance. DUF2213, a phage-associated domain (PF09979), is well distributed across bacteria and phages. Interestingly, we found that DUF4148 (PF13663) is putatively essential and associated with β-lactamase (PF00144) (Fig. 4).

In summary, our study identified 27 EDC genes whose knockdown produced a growth defect, suggesting the essential nature of one of their protein domains. By leveraging a Tn-Seq dataset in *B. cenocepacia* K56-2^17^, we demonstrate that the essential nature of protein-coding genes is a function of the individual protein domains they encode. The utility of our work lies in the identification of gene regions encoding essential and conserved protein domains, which will help de-orphan the many remaining proteins of unknown function. Therefore, we propose that determining essentiality of a domain of unknown function should be the first step in the process to define their function.

## Methods

### Bacterial strains and growth conditions

The list of bacterial strains and plasmids used in this study is provided in Supplementary Table 3. Bacterial strains were grown in LB-Lennox medium (Difco) at 37 °C. *E. coli* strain MM290 carrying the helper plasmid pRK2013 was selected in kanamycin 40 µg/mL (Fisher Scientific). Donor strains of *E. coli* DH5α and *B. cenocepacia* K56-2 carrying the sgRNA plasmids were selected in trimethoprim 50 µg/mL and 100 µg/mL (Sigma), respectively.

### Identification of EDC genes from Tn-Seq dataset

Candidate EDC genes were identified with a custom python script using the Tn-seq dataset^17^. The script analyzed every gene previously classified as “non-essential” by splitting it into two equal halves and counting the number of reads mapped to each half-gene. The script then used the “min ratio” and “min reads” as filtering criteria to call EDC genes. “Min ratio” was defined as the desired ratio of reads between the halves of the gene. “Min reads” was defined as the minimum number of reads in the non-empty end that is equal to a 14% of that half's length. Min reads was set to 0.14, while min ratio was set as 0. For each gene, 10% from each end of the gene was discarded from the analysis. The parameters can be changed to yield either more stringent or more general results. The script is available at https://github.com/cardonalab/EssentialDomains

### Bioinformatic analysis

Orthologous essential genes were identified using BLASTx against DEG 15^20^. Multidomain information was fetched from the UniProt database based on Pfam^8^ and InterPro^9^ domain features. DUF containing genes were characterized using the Pfam tool available on the Pfam website (https://pfam.xfam.org/). Domain sequences were retrieved in FASTA format from the Pfam database^8^ and aligned by Clustal Ω^39^. Maximum-likelihood phylogenetic trees were generated with MEGA-X^40^ using a Jones-Taylor-Thornton (JTT)-based model^41^ applying 100 bootstrap values. Phylogenetic trees were visualized, edited and taxonomic labels were assigned using Interactive Tree Of Life (i-TOL)^42^. Bootstrap values are represented on a scale of 0 to 1. Taxonomic annotations were labelled based on the NCBI taxonomy database using UniProt identifiers.

### Creating knockdown mutants of the candidate EDC genes with CRISPRi

CRISPRi mutants of the EDC genes were created as previously described^19^. Briefly, pSCB2-sgRNAv2, a modified plasmid from pSCB2-sgRNA^19^, was used as the template for inverse PCR to insert 20 bp target-specific sgRNA sequence. Inverse PCR was performed using Q5 high-fidelity polymerase (NEB), forward primers with individual sgRNAs as 5′ tail, and 1092 as the reverse primer. The resultant fragments were ligated to create circular plasmids by incubating 0.5µL of the respective PCR products with quick ligation buffer (NEB), 0.25 μL *DpnI*, 0.25 μL T4 polynucleotide kinase (NEB), and 0.25 μL T4 ligase (NEB) for 30 min at 37 °C. Resultant plasmids were transformed into *E. coli* DH5α, recovered for 2 h and selected in LB supplemented with trimethoprim 50 µg/mL (Sigma). The transformants were further confirmed by colony PCR using primers 1409 and 848. *E. coli* strains carrying the sgRNA plasmids were used as donors, and *E. coli* MM290/pRK2013 as the helper for triparental mating to introduce the sgRNA plasmids into *B. cenocepacia* K56-2 containing the chromosomally integrated dCas9 under the control of a rhamnose inducible promoter, as described previously^43^. Trimethoprim resistant colonies (100 µg/mL) were selected and screened by colony PCR using the primers 1409 and 848. The list of all the primers used in this study is provided in Supplementary Table 4.

### Conditional growth phenotype analysis of the CRISPRi mutants

To determine the conditional growth phenotype of the candidate genes, overnight cultures of the CRISPRi mutants were back diluted to OD_600nm_ 0.01. The cultures were grown at 37 °C for 20–24 h with continuous shaking in a 384-well plate containing LB broth supplemented with trimethoprim 100 µg/mL and with/without 1% rhamnose. OD_600nm_ readings were taken at 1-h intervals using BioTek Synergy 2 microplate reader.

## Supplementary Information

Supplementary Information.

Supplementary Table S1.

Supplementary Table S2.

Supplementary Tables.

Supplementary Table S5.
