# Supplementary material for: Identification of putative essential protein domains from high-density transposon insertion sequencing
Source: Sci Rep. 2022 Jan 19;12:962. doi: 10.1038/s41598-022-05028-x (PMC8770471; doi:10.1038/s41598-022-05028-x)
Supplement: Supplementary file 1 — Supplementary Information. [file 41598_2022_5028_MOESM1_ESM.pdf]

Identification of Putative Essential Protein Domains from High-density Transposon Insertion Sequencing

A. S. M. Zisanur Rahman[1], Lukas Timmerman[2], Flyn Gallardo[1], Silvia T. Cardona[1,3*]

[1]Department of Microbiology, University of Manitoba, Winnipeg, MB, Canada.

[2]Department of Computer Science, University of Manitoba, Winnipeg, MB, Canada.

[3]Department of Medical Microbiology & Infectious Diseases, University of Manitoba, Winnipeg, Canada.

*To whom correspondence should be addressed: Silvia.Cardona@umanitoba.ca

Supplemental Figures

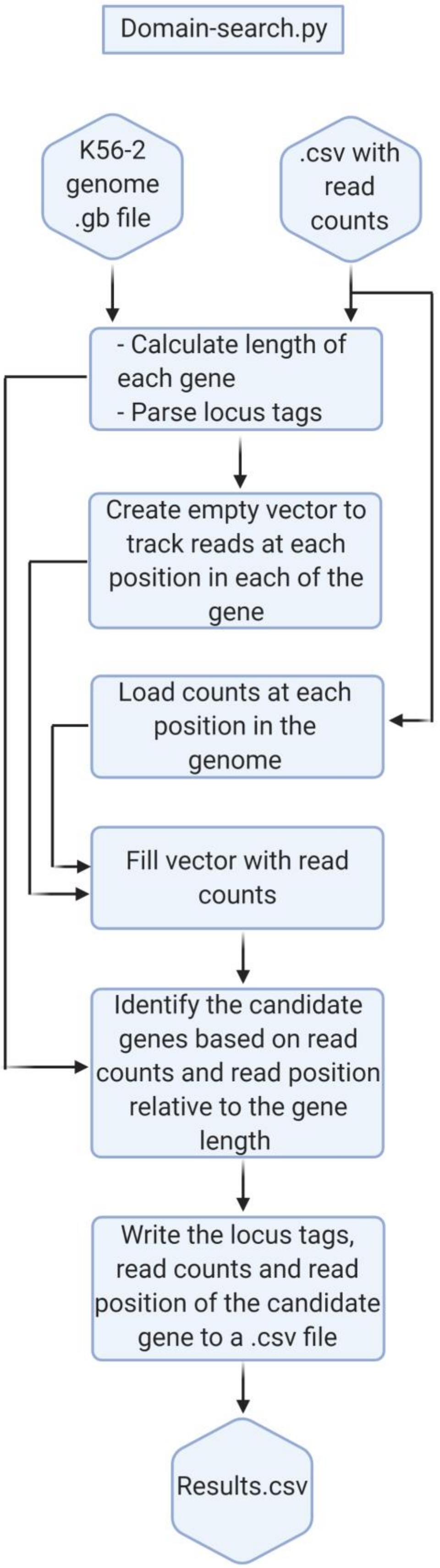
Domain-search.py
K56-2 genome .gb file
.csv with read counts
- Calculate length of each gene
- Parse locus tags
Create empty vector to track reads at each position in each of the gene
Load counts at each position in the genome
Fill vector with read counts
Identify the candidate genes based on read counts and read position relative to the gene length
Write the locus tags, read counts and read position of the candidate gene to a .csv file
Results.csv

Supplementary Figure 1: Workflow of the python script used to identify candidate essential domain-containing (EDC) genes in *B. cenocepacia* K56-2. The script used a csv file loaded with Tn-Seq read counts against each gene and a genbank file (.gb) of the *B. cenocepacia* K56-2 genome. First, the entire annotated genome of *B. cenocepacia* K56-2 was loaded, and the sequencing reads were mapped. Then, the insertion site counts from the Tn-Seq dataset were read and the number of insertions at each base pair of the genome was recorded. Finally, the script iterated over every gene and split the genes into two halves, calculating two parameters: min reads (the minimum number of reads to call out one end as non-essential). and 'min ratio' (the minimum ratio of reads between the two equal parts). The 'min reads' was set to 14% of the length of each half and 'min ratio' was set to 0.

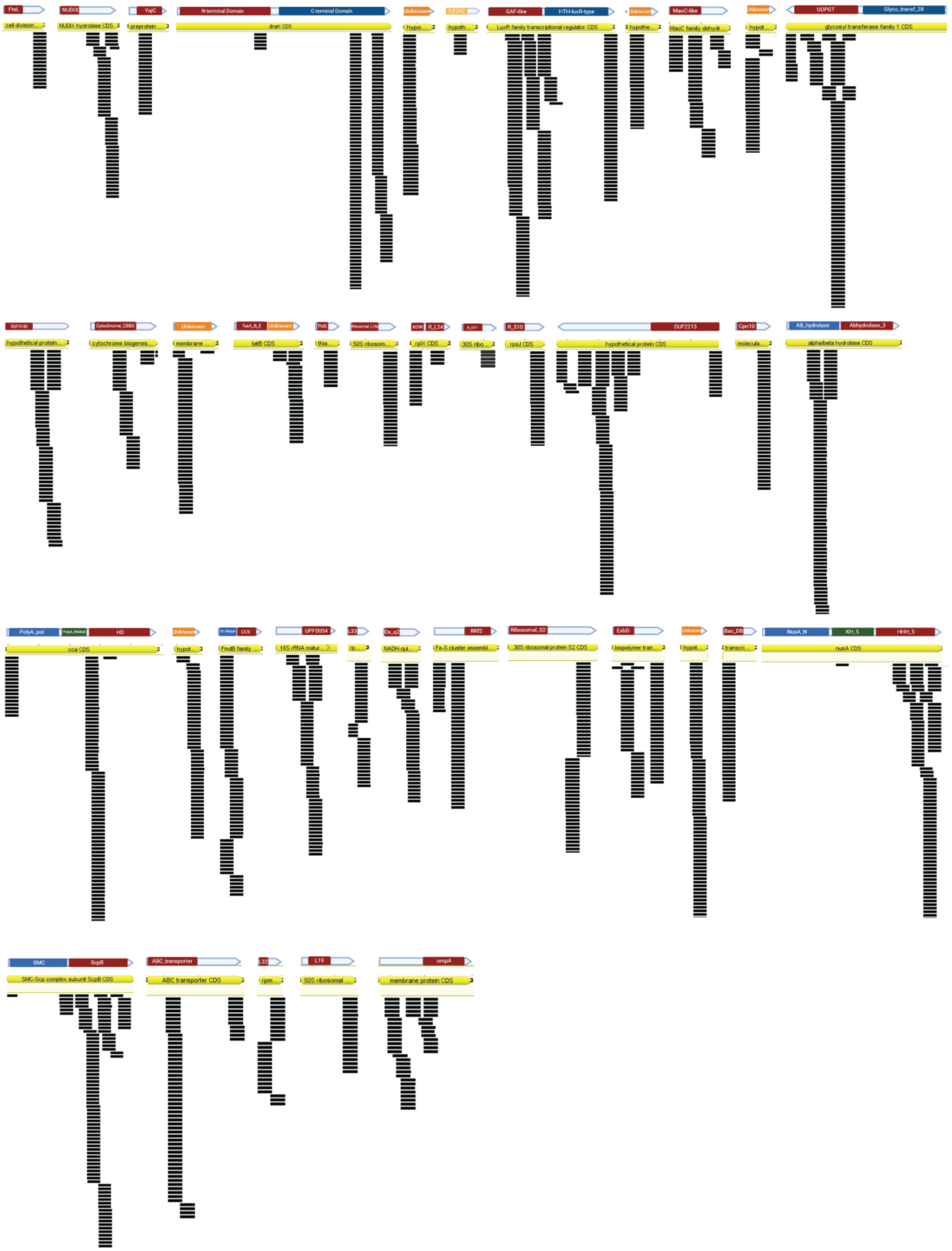

Supplementary Figure 2: Domain organization of the candidate EDC genes with Tn-Seq reads mapped to the corresponding genes. Tn-Seq reads were from [4] were mapped to the candidate EDC genes individually using Bowtie2 [5].

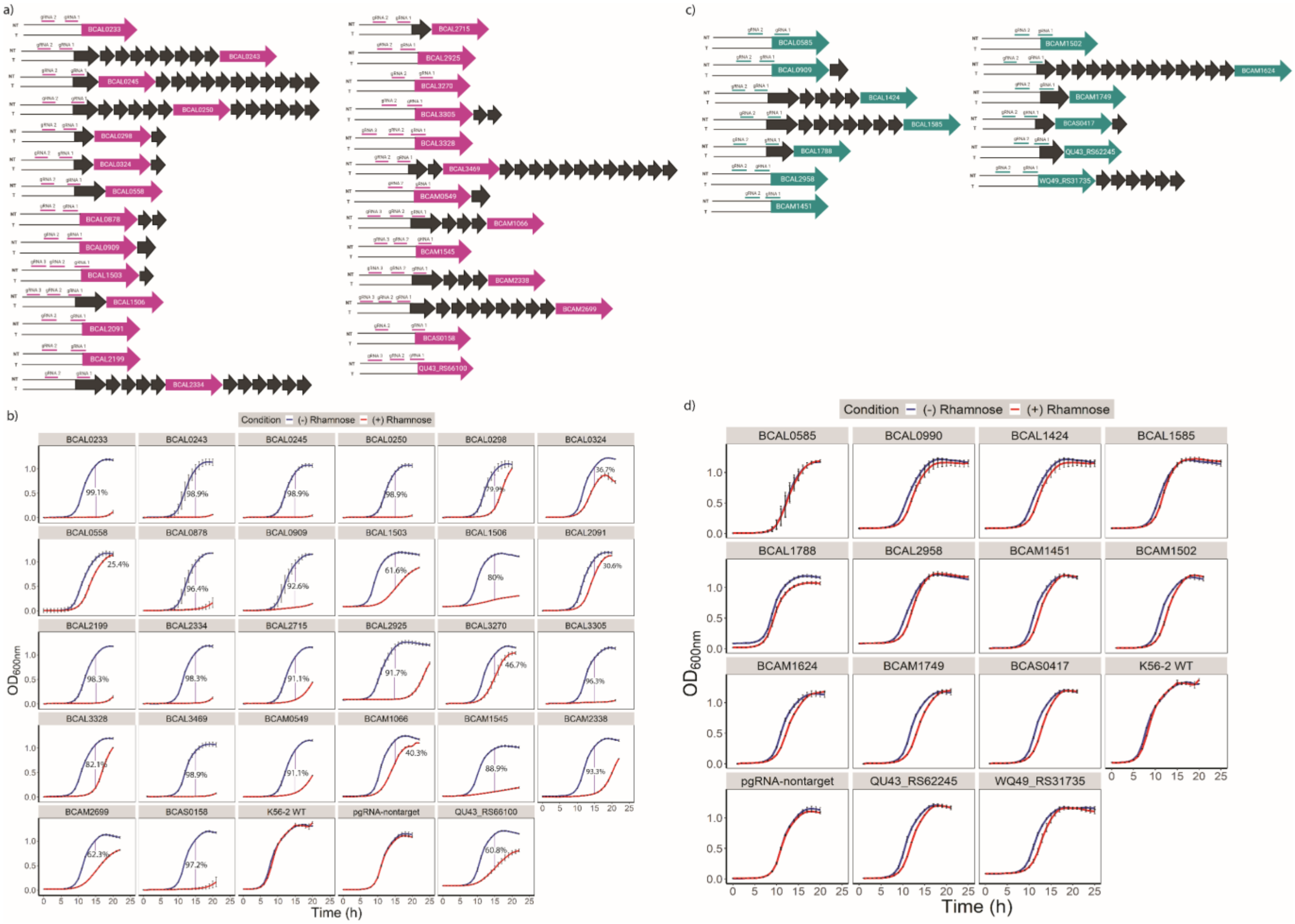

Supplementary Figure 3: CRISPRi mediated knockdown of the candidate genes resulted in growth defect in *B. cenocepacia* K56-2. Positions of the sgRNAs targeting candidate EDC genes that demonstrated growth defect **(a)** and genes that did not **(c)**. sgRNAs were designed to target a region close to the start codon and juxtapose to the putative transcription start site on the non-template strand (NT). Transcriptional inhibition of the candidate genes resulted in growth defect

in 27 candidate genes mutants. Percent numbers inside the plots indicate percent inhibition **(b)**. 13 genes did not exhibit any conditional growth phenotype **(d)**. Growth inhibition of at least 25% compared to the dCas9 uninduced condition was considered as growth defect. Overnight culture of the CRISPRi mutants, *B. cenocepacia* K56-2 wild-type (K56-2 WT) and a mutant harbouring non-genome targeting gRNA (K56-2/pgRNA-nontarget) were back diluted to OD_{600nm} of 0.01 and grown in LB supplemented with trimethoprim 100µg/mL for 20-24h with and without the dCas9 inducer, rhamnose. Results the average of three independent biological replicates. Error bars indicate mean ± SD.

References

1. Darling, P., Chan, M., Cox, A. D. & Sokol, P. A. Siderophore production by cystic fibrosis isolates of *Burkholderia cepacia*. *Infection and immunity* **66**, 874–877 (1998).
2. Hogan, A. M., Rahman, A. S. M. Z., Lightly, T. J. & Cardona, S. T. A Broad-Host-Range CRISPRi Toolkit for Silencing Gene Expression in Burkholderia. *ACS Synth Biol* **8**, 2372–2384 (2019).
3. Figurski, D. H. & Helinski, D. R. Replication of an origin-containing derivative of plasmid RK2 dependent on a plasmid function provided in trans. *Proceedings of the National Academy of Sciences of the United States of America* **76**, 1648–1652 (1979).
4. Gislason, A. S., Turner, K., Domaratzki, M. & Cardona, S. T. Comparative analysis of the *Burkholderia cenocepacia* K56-2 essential genome reveals cell envelope functions that are uniquely required for survival in species of the genus *Burkholderia*. *Microb Genom* **3**, e000140 (2017).
5. Langmead, B. & Salzberg, S. L. Fast gapped-read alignment with Bowtie 2. *Nature Methods* **9**, 357–359 (2012).
