# Supplementary material for: Identification of putative essential protein domains from high-density transposon insertion sequencing
Source: Sci Rep. 2022 Jan 19;12:962. doi: 10.1038/s41598-022-05028-x (PMC8770471; doi:10.1038/s41598-022-05028-x)
Supplement: Supplementary file 4 — Supplementary Tables. [file 41598_2022_5028_MOESM4_ESM.pdf]

Identification of Putative Essential Protein Domains from High-density Transposon Insertion Sequencing

A. S. M. Zisanur Rahman[1], Lukas Timmerman[2], Flyn Gallardo[1], Silvia T. Cardona[1,3*]

[1]Department of Microbiology, University of Manitoba, Winnipeg, MB, Canada.

[2]Department of Computer Science, University of Manitoba, Winnipeg, MB, Canada.

[3]Department of Medical Microbiology & Infectious Diseases, University of Manitoba, Winnipeg, Canada.

*To whom correspondence should be addressed: Silvia.Cardona@umanitoba.ca

Supplemental Tables 3 and 4

Supplemental Table 3. Bacterial strains and plasmids used in this work

Strain or Plasmid	Features	Source
Burkholderia cenocepacia K56-2	Clinical isolate from cystic fibrosis patient; ET12 lineage	[1]
K56-2::dCas9	Derived from K56-2; pAH-CTX1-rhadCas9 integrated at *attB* site; clean deletion of plasmid accessory genes	[2]
E. coli DH5α	F^- Φ80*lac*ZΔM15 Δ(*lac*ZYA-*arg*F) U169 *rec*A1 *end*A1 *hsd*R17 (r_H^+ , m_K^+) *pho*A *sup*E44 λ^- *thi*-1 *gyr*A96 *rel*A1	Cardona lab collection
E. coli MM290	F-, φ 80 lacZΔM15 *endA1 recA1 hsdR17* (r_H^- m_K^+) *supE*44 *thi*-1 Δ*gyrA96* (*ΔlacZYA*-*arg*F) U169 *rel*A1	Cardona lab collection
pSCB2-sgRNAv2	Derived from pSCB2-sgRNA; modified by inserting a BamHI site downstream of the sgRNA scaffold	This study; modified from [2]
pgRNA-nontarget	Derived from pSCB2-sgRNA; random 20nt sequence added as sgRNA binding region by inverse PCR	[2]
pRK2013	Ori_{colE1} RK2 derivative Kan^r mob^+ tra^+	[3]

Supplemental Table 4. List of the primers used in this study

Primer Number	Sequence	Purpose
848	CCGCCAGGCAAATTCTGTTT	Reverse primer for colony PCR, all sgRNAs
1092	ACTAGTATTATACCTAGGACTGAGCTAGC	Reverse primer, all sgRNAs
1409	GGCTTATGTCAACTGGGTTCG	Forward primer for colony PCR, all sgRNAs
1685	CAGTTGCACAGTGTCCTCTCGTTTTAGAG CTAGAAATAGCAAGTTAAAATAAGGC	Forward primer, sgRNA 1 for targeting WQ49_RS32210 (BCAL1506) in K56-2
1686	AGAGTTCGGGTTTCATGGTTGTTTTAGAG CTAGAAATAGCAAGTTAAAATAAGGC	Forward primer, sgRNA 1 for targeting WQ49_RS00770 (BCAL3328) in K56-2
1687	GCGGGGGCGGATGGCATCGCGTTTTAGA GCTAGAAATAGCAAGTTAAAATAAGGC	Forward primer, sgRNA 1 for targeting WQ49_RS03390 (BCAM1545) in K56-2
1688	CGGCGGAATGAAGGTTGTCGGTTTTAGA GCTAGAAATAGCAAGTTAAAATAAGGC	Forward primer, sgRNA 1 for targeting WQ49_RS07360 (BCAM2338) in K56-2
1689	CTGCCCCCTCTCTTCACAAAGTTTTAGAG CTAGAAATAGCAAGTTAAAATAAGGC	Forward primer, sgRNA 1 for targeting WQ49_RS07395 (QU43_RS66100) in K56-2
1690	CCGCGTTGCGCGGCGAGCGGGTTTTAGA GCTAGAAATAGCAAGTTAAAATAAGGC	Forward primer, sgRNA 1 for targeting WQ49_RS16145 (BCAM1066) in K56-2
1691	TCTTGGGTATTCATGCAGCTGTTTTAGAG CTAGAAATAGCAAGTTAAAATAAGGC	Forward primer, sgRNA 1 for targeting WQ49_RS32225 (BCAL1503) in K56-2
1692	TCTCCGTGTGTTCTGTGCCGGTTTTAGAG CTAGAAATAGCAAGTTAAAATAAGGC	Forward primer, sgRNA 1 for targeting WQ49_RS22170 (BCAM2699) in K56-2
1693	CCGCGGGCGCGCGCCTTAGCGTTTTAGA GCTAGAAATAGCAAGTTAAAATAAGGC	Forward primer, sgRNA 2 for targeting WQ49_RS32210 (BCAL1506) in K56-2

1694	CCCGAACGCTGCCGGCCGTCGTTTTAGAG CTAGAAATAGCAAGTTAAAATAAGGC	Forward primer, sgRNA 2 for targeting WQ49_RS00770 (BCAL3328) in K56-2
1695	TCGTCTCCTGTCGCGTCGTCGTTTTAGAG CTAGAAATAGCAAGTTAAAATAAGGC	Forward primer, sgRNA 2 for targeting WQ49_RS03390 (BCAM1545) in K56-2
1696	TAACGATCAGACGATCGTGCGTTTTAGA GCTAGAAATAGCAAGTTAAAATAAGGC	Forward primer, sgRNA 2 for targeting WQ49_RS07360 (BCAM2338) in K56-2
1697	CAGGAACGCGACCGTCCGGCGTTTTAGA GCTAGAAATAGCAAGTTAAAATAAGGC	Forward primer, sgRNA 2 for targeting WQ49_RS07395 (QU43_RS66100) in K56-2
1698	TGTTCGTGCCGGTCTATTCCGTTTTAGAG CTAGAAATAGCAAGTTAAAATAAGGC	Forward primer, sgRNA 2 for targeting WQ49_RS16145 (BCAM1066) in K56-2
1699	CGGATCAACAAACAGACGAAGTTTTAGA GCTAGAAATAGCAAGTTAAAATAAGGC	Forward primer, sgRNA 2 for targeting WQ49_RS32225 (BCAL1503) in K56-2
1700	GAAGCCTAGTATCGTCACGAGTTTTAGA GCTAGAAATAGCAAGTTAAAATAAGGC	Forward primer, sgRNA 2 for targeting WQ49_RS22170 (BCAM2699) in K56-2
1701	GGAACGCCCATCTTCTTACTGTTTTAGAG CTAGAAATAGCAAGTTAAAATAAGGC	Forward primer, sgRNA 3 for targeting WQ49_RS32210 (BCAL1506) in K56-2
1702	CCCCGGCAGATCGTCCGTGCGTTTTAGAG CTAGAAATAGCAAGTTAAAATAAGGC	Forward primer, sgRNA 3 for targeting WQ49_RS00770 (BCAL3328) in K56-2
1703	TGGTCGGCGCCGCCGTTTGTGTTTTAGAG CTAGAAATAGCAAGTTAAAATAAGGC	Forward primer, sgRNA 3 for targeting WQ49_RS03390 (BCAM1545) in K56-2
1704	ATCGGCGACGCGCTCCGCGCGTTTTAGA GCTAGAAATAGCAAGTTAAAATAAGGC	Forward primer, sgRNA 3 for targeting WQ49_RS07360 (BCAM2338) in K56-2
1705	CCGGCACGCGTCATGCGCGCGTTTTAGA GCTAGAAATAGCAAGTTAAAATAAGGC	Forward primer, sgRNA 3 for targeting

		WQ49_RS07395 (QU43_RS66100) in K56-2
1706	CCCGGAGGACGACCAGCTCCGTTTTAGA GCTAGAAATAGCAAGTTAAAATAAGGC	Forward primer, sgRNA 3 for targeting WQ49_RS16145 (BCAM1066) in K56-2
1707	ATTCTGATCGGTCATATTGAGTTTTAGAG CTAGAAATAGCAAGTTAAAATAAGGC	Forward primer, sgRNA 3 for targeting WQ49_RS32225 (BCAL1503) in K56-2
1708	GAAATTCCGTTCCACATACGGTTTTAGAG CTAGAAATAGCAAGTTAAAATAAGGC	Forward primer, sgRNA 3 for targeting WQ49_RS22170 (BCAM2699) in K56-2
1742	CACTTTCCGCTCTCCCGTTCGTTTTAGAG CTAGAAATAGCAAGTTAAAATAAGGC	Forward primer, sgRNA 1 for targeting WQ49_RS00050 (BCAL3469) in K56-2
1743	CTCAATGCTGAGAATTTAGCGTTTTAGAG CTAGAAATAGCAAGTTAAAATAAGGC	Forward primer, sgRNA 2 for targeting WQ49_RS00050 (BCAL3469) in K56-2
1744	CGGCACGTTGGTCTCTCCGTGTTTTAGAG CTAGAAATAGCAAGTTAAAATAAGGC	Forward primer, sgRNA 1 for targeting WQ49_RS00885 (BCAL3305) in K56-2
1745	TAAGCCGGTTATTCTACCACGTTTTAGAG CTAGAAATAGCAAGTTAAAATAAGGC	Forward primer, sgRNA 2 for targeting WQ49_RS00885 (BCAL3305) in K56-2
1746	CGCACGAGTTCGTGGTGCCGGTTTTAGAG CTAGAAATAGCAAGTTAAAATAAGGC	Forward primer, sgRNA 1 for targeting WQ49_RS01035 (BCAL3270) in K56-2
1747	TGCGCGAAACGCCGCGTTTTGTTTTAGAG CTAGAAATAGCAAGTTAAAATAAGGC	Forward primer, sgRNA 2 for targeting WQ49_RS01035 (BCAL3270) in K56-2
1748	GGTTTCATTTGAGTTCTCCTGTTTTAGAG CTAGAAATAGCAAGTTAAAATAAGGC	Forward primer, sgRNA 1 for targeting WQ49_RS02920 (BCAM1451) in K56-2
1749	TGCCAGTCTCGCTGGCAGTGGTTTTAGAG CTAGAAATAGCAAGTTAAAATAAGGC	Forward primer, sgRNA 2 for targeting WQ49_RS02920 (BCAM1451) in K56-2
1750	AAATTGTCCTTGGACATCGAGTTTTAGAG CTAGAAATAGCAAGTTAAAATAAGGC	Forward primer, sgRNA 1 for targeting WQ49_RS03160 (BCAM1502) in K56-2
1751	TTAAAGACTAGACAGATACGGTTTTAGA GCTAGAAATAGCAAGTTAAAATAAGGC	Forward primer, sgRNA 2 for targeting WQ49_RS03160 (BCAM1502) in K56-2
1752	GAATTTCCTGTCCATGGCATGTTTTAGAG CTAGAAATAGCAAGTTAAAATAAGGC	Forward primer, sgRNA 1 for targeting WQ49_RS03550 (QU43_RS62245) in K56-2

1753	GCGCAACCGGTACGCGGCCGGTTTTAGA GCTAGAAATAGCAAGTTAAAATAAGGC	Forward primer, sgRNA 2 for targeting WQ49_RS03550 (QU43_RS62245) in K56-2
1754	CGCACGGACATCGATCATGTGTTTTAGAG CTAGAAATAGCAAGTTAAAATAAGGC	Forward primer, sgRNA 1 for targeting WQ49_RS03805 (BCAM1624) in K56-2
1755	AAAGGATTCTGGCCGGTCGGGTTTTAGA GCTAGAAATAGCAAGTTAAAATAAGGC	Forward primer, sgRNA 2 for targeting WQ49_RS03805 (BCAM1624) in K56-2
1756	AGGCAGGCGCGCGATGAGGCGTTTTAGA GCTAGAAATAGCAAGTTAAAATAAGGC	Forward primer, sgRNA 1 for targeting WQ49_RS04450 (BCAM1749) in K56-2
1757	GGCTGCGCGGAATGCGTGGGGTTTTAGA GCTAGAAATAGCAAGTTAAAATAAGGC	Forward primer, sgRNA 2 for targeting WQ49_RS04450 (BCAM1749) in K56-2
1758	CGGTCATGGTCGAACGCGTGGTTTTAGA GCTAGAAATAGCAAGTTAAAATAAGGC	Forward primer, sgRNA 1 for targeting WQ49_RS09185 (BCAS0417) in K56-2
1759	AAACGATCATTCTCGGCAACGTTTTAGAG CTAGAAATAGCAAGTTAAAATAAGGC	Forward primer, sgRNA 2 for targeting WQ49_RS09185 (BCAS0417) in K56-2
1760	GAAACGAGCGTTTTCATCATGTTTTAGAG CTAGAAATAGCAAGTTAAAATAAGGC	Forward primer, sgRNA 1 for targeting WQ49_RS10495 (BCAS0158) in K56-2
1761	GTTGCGCGTGCAGCCAATATGTTTTAGAG CTAGAAATAGCAAGTTAAAATAAGGC	Forward primer, sgRNA 2 for targeting WQ49_RS10495 (BCAS0158) in K56-2
1762	TCCTCAACCACGCCGCGTCGGTTTTAGAG CTAGAAATAGCAAGTTAAAATAAGGC	Forward primer, sgRNA 1 for targeting WQ49_RS11915 (BCAL0324) in K56-2
1763	GCCGGCTTCGACTATCGATGGTTTTAGAG CTAGAAATAGCAAGTTAAAATAAGGC	Forward primer, sgRNA 2 for targeting WQ49_RS11915 (BCAL0324) in K56-2
1764	TTCATCGCGCGCCCTCCGCGGTTTTAGAG CTAGAAATAGCAAGTTAAAATAAGGC	Forward primer, sgRNA 1 for targeting WQ49_RS12045 (BCAL0298) in K56-2
1765	CATGCGAACGCACGCGACATGTTTTAGA GCTAGAAATAGCAAGTTAAAATAAGGC	Forward primer, sgRNA 2 for targeting WQ49_RS12045 (BCAL0298) in K56-2
1766	CCGAGATTCGGTCTGGATCAGTTTTAGAG CTAGAAATAGCAAGTTAAAATAAGGC	Forward primer, sgRNA 1 for targeting WQ49_RS12280 (BCAL0250) and WQ49_RS12305 (BCAL0245) in K56-2
1767	ATGCACCACCATGATGCATTGTTTTAGAG CTAGAAATAGCAAGTTAAAATAAGGC	Forward primer, sgRNA 2 for targeting WQ49_RS12280

		(BCAL0250) and WQ49_RS12305 (BCAL0245) in K56-2
1768	TCTCCATTCCCGACTGCGATGTTTTAGAG CTAGAAATAGCAAGTTAAAATAAGGC	Forward primer, sgRNA 1 for targeting WQ49_RS12315 (BCAL0243) in K56-2
1769	TACAAAATGCCGGGCACGCAGTTTTAGA GCTAGAAATAGCAAGTTAAAATAAGGC	Forward primer, sgRNA 2 for targeting WQ49_RS12315 (BCAL0243) in K56-2
1770	ACAATTCCTTGAAAAGAGCGGTTTTAGA GCTAGAAATAGCAAGTTAAAATAAGGC	Forward primer, sgRNA 1 for targeting WQ49_RS12365 (BCAL0233) in K56-2
1771	ACGTGAAACATGACACCAGCGTTTTAGA GCTAGAAATAGCAAGTTAAAATAAGGC	Forward primer, sgRNA 2 for targeting WQ49_RS12365 (BCAL0233) in K56-2
1772	CGCGATCGTGCAAGGGGCGTGTTTTAGA GCTAGAAATAGCAAGTTAAAATAAGGC	Forward primer, sgRNA 1 for targeting WQ49_RS18705 (BCAM0549) in K56-2
1773	AGTGCTGACGAGTATAAAAAGTTTTAGA GCTAGAAATAGCAAGTTAAAATAAGGC	Forward primer, sgRNA 2 for targeting WQ49_RS18705 (BCAM0549) in K56-2
1774	CAATGACGAGCTTCATGACGGTTTTAGA GCTAGAAATAGCAAGTTAAAATAAGGC	Forward primer, sgRNA 1 for targeting WQ49_RS23945 (BCAL0558) in K56-2
1775	CGAAAAAGGAAAAGAAGCGCGTTTTAGA GCTAGAAATAGCAAGTTAAAATAAGGC	Forward primer, sgRNA 2 for targeting WQ49_RS23945 (BCAL0558) in K56-2
1776	CGATTTCACCCACGACGTTCGTTTTAGAG CTAGAAATAGCAAGTTAAAATAAGGC	Forward primer, sgRNA 1 for targeting WQ49_RS24070 (BCAL0585) in K56-2
1777	CGGACCGTGTTTGTCATCGCGTTTTAGAG CTAGAAATAGCAAGTTAAAATAAGGC	Forward primer, sgRNA 2 for targeting WQ49_RS24070 (BCAL0585) in K56-2
1778	TTTCCCGCAGAAACTGGAAAGTTTTAGA GCTAGAAATAGCAAGTTAAAATAAGGC	Forward primer, sgRNA 1 for targeting WQ49_RS25525 (BCAL0878) in K56-2
1779	TTGAATTATAGCCGAAACCCGTTTTAGAG CTAGAAATAGCAAGTTAAAATAAGGC	Forward primer, sgRNA 2 for targeting WQ49_RS25525 (BCAL0878) in K56-2
1780	GGAGGATTTCATGACGCGTGGTTTTAGA GCTAGAAATAGCAAGTTAAAATAAGGC	Forward primer, sgRNA 1 for targeting WQ49_RS25680 (BCAL0909) in K56-2
1781	CGGCCGATCGCGCCCGAATTGTTTTAGAG CTAGAAATAGCAAGTTAAAATAAGGC	Forward primer, sgRNA 2 for targeting WQ49_RS25680 (BCAL0909) in K56-2

1782	CTCCTAATACGCTAAATTCGGTTTTAGAG CTAGAAATAGCAAGTTAAAATAAGGC	Forward primer, sgRNA 1 for targeting WQ49_RS26625 (BCAL2715) in K56-2
1783	TCGCACCGCCACGTGATCCAGTTTTAGAG CTAGAAATAGCAAGTTAAAATAAGGC	Forward primer, sgRNA 2 for targeting WQ49_RS26625 (BCAL2715) in K56-2
1784	ACGCTCGATCGATTTCCTTCGTTTTAGAG CTAGAAATAGCAAGTTAAAATAAGGC	Forward primer, sgRNA 1 for targeting WQ49_RS27920 (BCAL2334) in K56-2
1785	ACGAACAACCTGCCGCACACGTTTTAGA GCTAGAAATAGCAAGTTAAAATAAGGC	Forward primer, sgRNA 2 for targeting WQ49_RS27920 (BCAL2334) in K56-2
1786	TGGTGAGTCTCATGGTGTCTGTTTTAGAG CTAGAAATAGCAAGTTAAAATAAGGC	Forward primer, sgRNA 1 for targeting WQ49_RS28635 (BCAL2199) in K56-2
1787	AAGTATCCATGTCTTTGAAAGTTTTAGAG CTAGAAATAGCAAGTTAAAATAAGGC	Forward primer, sgRNA 2 for targeting WQ49_RS28635 (BCAL2199) in K56-2
1788	TGTAAATTCTCCACGAGGGTGTTTTAGAG CTAGAAATAGCAAGTTAAAATAAGGC	Forward primer, sgRNA 1 for targeting WQ49_RS29230 (BCAL2091) in K56-2
1789	GCCGCGCGAACCCGCCACGAGTTTTAGA GCTAGAAATAGCAAGTTAAAATAAGGC	Forward primer, sgRNA 2 for targeting WQ49_RS29230 (BCAL2091) in K56-2
1790	CAACAGGCTTGCCGCCAGCGGTTTTAGA GCTAGAAATAGCAAGTTAAAATAAGGC	Forward primer, sgRNA 1 for targeting WQ49_RS30770 (BCAL1788) in K56-2
1791	CAAGCCGGTTTTCACCGGTCGTTTTAGAG CTAGAAATAGCAAGTTAAAATAAGGC	Forward primer, sgRNA 2 for targeting WQ49_RS30770 (BCAL1788) in K56-2
1792	TCGTTATCCATCAACAGAAAGTTTTAGAG CTAGAAATAGCAAGTTAAAATAAGGC	Forward primer, sgRNA 1 for targeting WQ49_RS31735 in K56-2
1793	CGCCACAAAACCGCGCTGCCGTTTTAGA GCTAGAAATAGCAAGTTAAAATAAGGC	Forward primer, sgRNA 2 for targeting WQ49_RS31735 in K56-2
1794	TTCGCGCAGCTTGCGCTTGAGTTTTAGAG CTAGAAATAGCAAGTTAAAATAAGGC	Forward primer, sgRNA 1 for targeting WQ49_RS31805 (BCAL1585) in K56-2
1795	GCACAATAGCAAACAAACGTGTTTTAGA GCTAGAAATAGCAAGTTAAAATAAGGC	Forward primer, sgRNA 2 for targeting WQ49_RS31805 (BCAL1585) in K56-2
1796	GATGAGAATCTGGCGAGCGCGTTTTAGA GCTAGAAATAGCAAGTTAAAATAAGGC	Forward primer, sgRNA 1 for targeting WQ49_RS32625 (BCAL1424) in K56-2

1797	CGGCTGCCCGCGCACCCGCTGTTTTAGAG CTAGAAATAGCAAGTTAAAATAAGGC	Forward primer, sgRNA 2 for targeting WQ49_RS32625 (BCAL1424) in K56-2
1798	TTATGCCATCGTGTCGGCGTGTTTTAGAG CTAGAAATAGCAAGTTAAAATAAGGC	Forward primer, sgRNA 1 for targeting WQ49_RS34895 (BCAL2925) in K56-2
1799	TCCAGGCCTGGCCCCGATAGGTTTTAGAG CTAGAAATAGCAAGTTAAAATAAGGC	Forward primer, sgRNA 2 for targeting WQ49_RS34895 (BCAL2925) in K56-2
1800	CTCGAAATTGAGATTACCGCGTTTTAGAG CTAGAAATAGCAAGTTAAAATAAGGC	Forward primer, sgRNA 1 for targeting WQ49_RS35060 (BCAL2958) in K56-2
1801	CATTGCTCGAAGTATAACATGTTTTAGAG CTAGAAATAGCAAGTTAAAATAAGGC	Forward primer, sgRNA 2 for targeting WQ49_RS35060 (BCAL2958) in K56-2
